# A study on stress distribution to cement layer and root dentin for post and cores made of CAD/CAM materials with different elasticity modulus in the absence of ferrule

**DOI:** 10.4317/jced.55295

**Published:** 2019-01-01

**Authors:** Guilherme -Schmidt de Andrade, João-Paulo-Mendes Tribst, Amanda-Maria-de Oliveira Dal Piva, Marco-Antonio Bottino, Alexandre-Luiz-Souto Borges, Luiz-Felipe Valandro, Mutlu Özcan

**Affiliations:** 1 DDs, MSc Student in Prosthodontics, Department of Dental Materials and Proshodontics, São Paulo State University (Unesp), Institute of Science and Technology, São José dos Campos / SP, Brazil. Address: Av Engenheiro Francisco José Longo, 777, Jardim São Dimas, São José dos Campos, São Paulo, Brazil; 2 DDs, MSc, PhD Student, Department of Dental Materials and Proshodontics, São Paulo State University (Unesp), Institute of Science and Technology, São José dos Campos / SP, Brazil. Address: Av Engenheiro Francisco José Longo, 777, Jardim São Dimas, São José dos Campos, São Paulo, Brazil; 3 DDs, MSc, PhD, Professor, Department of Dental Materials and Proshodontics, São Paulo State University (Unesp), Institute of Science and Technology, São José dos Campos / SP, Brazil. Address: Av Engenheiro Francisco José Longo, 777, Jardim São Dimas, São José dos Campos, São Paulo, Brazil; 4 DDs, MSc, PhD, Adjunct Professor, Department of Dental Materials and Proshodontics, São Paulo State University (Unesp), Institute of Science and Technology, São José dos Campos / SP, Brazil. Address: Av Engenheiro Francisco José Longo, 777, Jardim São Dimas, São José dos Campos, São Paulo, Brazil; 5 DDs, MSc, PhD, Professor, Federal University of Santa Maria, Prosthodontic Unit, Faculty of Odontology, Santa Maria, Rio Grande do Sul State, Brazil; 6 DDs, MSc, PhD, Professor, University of Zurich, Dental Materials Unit, Center for Dental and Oral Medicine, Clinic for Fixed and Removable Prosthodontics and Dental Materials Science, Zurich, Switzerland

## Abstract

**Background:**

To evaluate the stress distribution in a maxillary central incisor with different post and cores made of six CAD/CAM materials with different elastic modulus in the absence of ferrule using the finite element analysis.

**Material and Methods:**

A three-dimensional endodontically treated maxillary central incisor restored with an all-ceramic crown was modelled in Rhinoceros (5.0 SR8, McNeel). The geometries were analyzed in ANSYS 17.2 (ANSYS Inc.) considering isotropic, homogeneous, linearly elastic materials with perfectly bonded contacts. The elastic moduli (E) of the post-and-cores defined the groups to be compared: nanoceramic resin (E=12.8GPa); composite resin (E=16GPa); hybrid ceramic (E=34.7GPa); lithium disilicate (E=95GPa); titanium (Ti-Al6-V4) (E=112GPa); and Y-TZP material (E=209.3GPa). The set was constrained in the cortical bone and loaded (45°/100 N) on the incisor palatine face. Stress distribution was analyzed by Maximum Principal Stress criteria for the crown-core cement line, Post-and-core’s cement line, Post-and-core system and Dentin.

**Results:**

The stress distribution at the crown-core cement line (11.4 – 13.2 MPa) was inversely proportional to the increase of the elastic modulus of the post-core approaches, while it was direct proportional on the post-and-core (4.7 – 40 MPa) and cement line (4.1 – 6.2 MPa). Stress distribution on the dentin was similar for all groups (24.7 - 25.3).

**Conclusions:**

Post-and-core made by CAD/CAM seems to be an efficient treatment alternative, since it is a conservative approach, promotes better aesthetic quality and it allows the control of the cement line thickness.

** Key words:**Endodontically treated teeth, Post-and-core technique, Ceramic crown, Finite element analysis, Biomimetics.

## Introduction

Posts are normally used to provide retention to the core in endodontically treated teeth with extensive loss of coronal and intracanal structures. In these cases, cast post and cores in gold alloys have been considered the gold standard due to their excellent success rate, favorable long-term prognosis and ease of manufacturing (1).

However, there is a concern as to how much the core color would affect the aesthetic outcome of the final restoration (2), since over the years the use of all-ceramic restorations has been extensively increasing, and a plausible consequence might be the oxidation effect of some metallic alloys over the gingival margin in a thin periodontium (3).

Thus, prefabricated resin-reinforced glass fiber posts associated with direct composite resin cores have been used as an aesthetic alternative when restoring anterior teeth (4). It is argued that these posts have an elastic modulus closer to the dentin, which provides a uniform stress distribution on the post/cement/dentin interfaces and on the dental remnant structure under masticatory intermittent loading, thus minimizing the risk of catastrophic root fracture (5). However, the prefabricated posts associated with direct cores when exposed to the clinical intermittent cyclic loading are subject to gaps or debonding of the post/core interface, increasing the failure potential of this system over the clinical service (for instance, loss of retention of the assembly) (6). Because they are prefabricated, such posts might not adequately adapt to the anatomy or specific conditions of root canals (oval shape, flared roots), resulting in a larger cement line which might cause an increase in the risk of loss of post retention (7,8). The thickness of the resin cement that shows the best stress distribution is up to 0.3 mm (7). On the other hand, customized post and cores could be an option for canals in which a prefabricated post could not adapt properly (reduction of cement’s thickness).

The evolution of CAD/CAM systems and the availability of ceramic blocks with superior aesthetic characteristics such as translucency and color gradient allow the preparation of monolithic restorations with a satisfactory aesthetic result. The industrial processing of these ceramic blocks results in higher structural reliability (reduction of defects population into the material bulk) (9).

In addition, it is known that the fracture strength and fatigue resistance of all-ceramic restorations is increased when adhesively luted in a susceptible substrate (10), which does not occur under cast metal cores since these materials have low adhesive characteristics.

Currently CAD/CAM systems allow machining of a wide range of dental materials, such as the glass-ceramics (feldspathic, leucite-reinforced feldspar, and lithium disilicate), laboratory resins, yttria stabilized zirconia, metals, and more recently the polymer infiltrated ceramic (PIC material) or also known as hybrid ceramics (9). This allows the clinician to choose the best material according to the need of each clinical case (11). In addition, these systems allow the restoration to be designed by a particular software, which gives the dentist better control of the characteristics of the restorations, such as controlling the thickness, shape, marginal features, occlusion of the restoration, as well as, thickness of the resin cement.

In terms of root restoration, that restoring approach permit to control the resin cement’s thickness when restoring oval root canals and flared canals, different from inherent circumstances for pre-fabricated post (thicker resin cement), which might influence the stress distribution on the restorative assembly. Besides, materials with distinct elastic modulus milled by CAD/CAM system might affect the stress distribution on root/assembly, influencing the clinical performance overtime.

Thus, this current study aimed to assess the stress distribution (biomechanical behavior) in a maxillary central incisor with different post and cores made of six CAD/CAM materials with different elastic modulus through finite element analysis. The null hypotheses tested were that the elastic modulus of the material does not affect stress distribution at the: 1) cement line between the full crown and the core build-up; 2) cement line between the post and the root; 3) post; or 4) remaining dentin.

## Material and Methods

-Finite Element analysis for pre-processing

**Figure 1:**
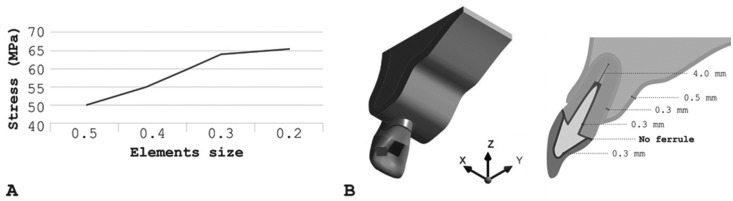
(A-B) - Schematic illustration of modeling parameters. A) Graph of mesh convergence test, B) Central incisor model subjected to oblique force (45°) on palatal surface. The red square shows the point of load application.

The 3D model used in a previous study (7) composed of periodontal ligament and cortical bone with an 0.3 and 0.5 mm thickness, respectively, medullary bone, gutta percha with length of 4 mm, post and core, all-ceramic crown of lithium disilicate (e.max CAD, Ivoclar Vivadent, Schaan, Liechtenstein), and dentin were prepared for full-crown without ferrule. Both the crown and the post and core were cemented with a 0.3 mm (Fig. 1) line of resin cement Panavia F (Kuraray, Tokyo, Japan).

-Finite element analysis for processing

The geometries were imported in STEP format to Computer Aided Engineering (CAE) software (ANSYS 17.2, ANSYS Inc.), where meshes were generated through the convergence test until obtaining a number of nodes (382,383) and elements (215,828) unable to interfere with the study outcome (Fig. 1A). The materials were considered isotropic, homogeneous and linearly elastic with perfectly bonded contacts. The groups were divided according to the elastic modulus of the materials used for restoring the post-and-core ( Table 1) (12-17): Nanoceramic resin (Lava Ultimate, 3M ESPE, St. Paul, MN, USA); composite resin (Paradigm MZ10, 3M ESPE); hybrid ceramic (Enamic, VITA Zahnfabrik, Bad Säckingen, BW, Germany); lithium disilicate (e-max CAD, Ivoclar Vivadent); titanium (Ti-Al6-V4) and tetragonal zirconia polycrystalline stabilized by yttria (Y-TZP, VITA Zahnfabrik). An oblique (45°) load of 100 N(7) was applied to the central region of the palatine face (Fig. 1B), with the system fixated at the cortical bone base. In this study, the stress distribution was analyzed by the Maximum Principal Stress criteria.

**Table 1:**
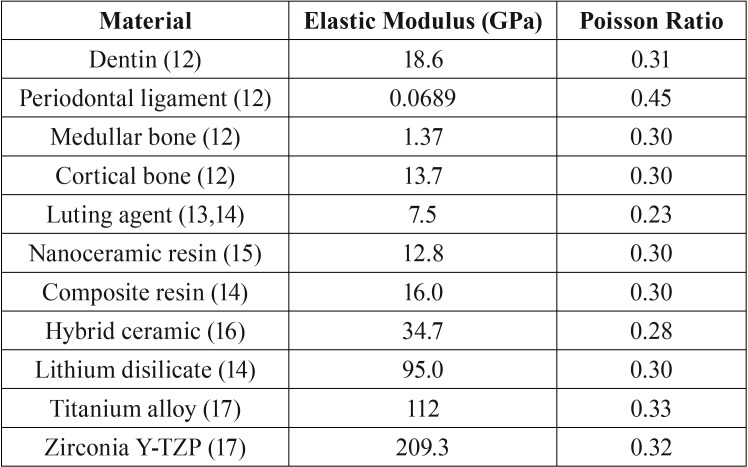
Properties of materials used in models.

-Analysis

The data obtained from the stress distribution at the cement line between crown and core, between post and root dentin, post and dentin were analyzed qualitatively and quantitatively through graphs and tables using the 10% confidence limit established by the mesh convergence test. The maximum stresses were compared with data from laboratory tests already published in the literature.

## Results

**Figure 2:**
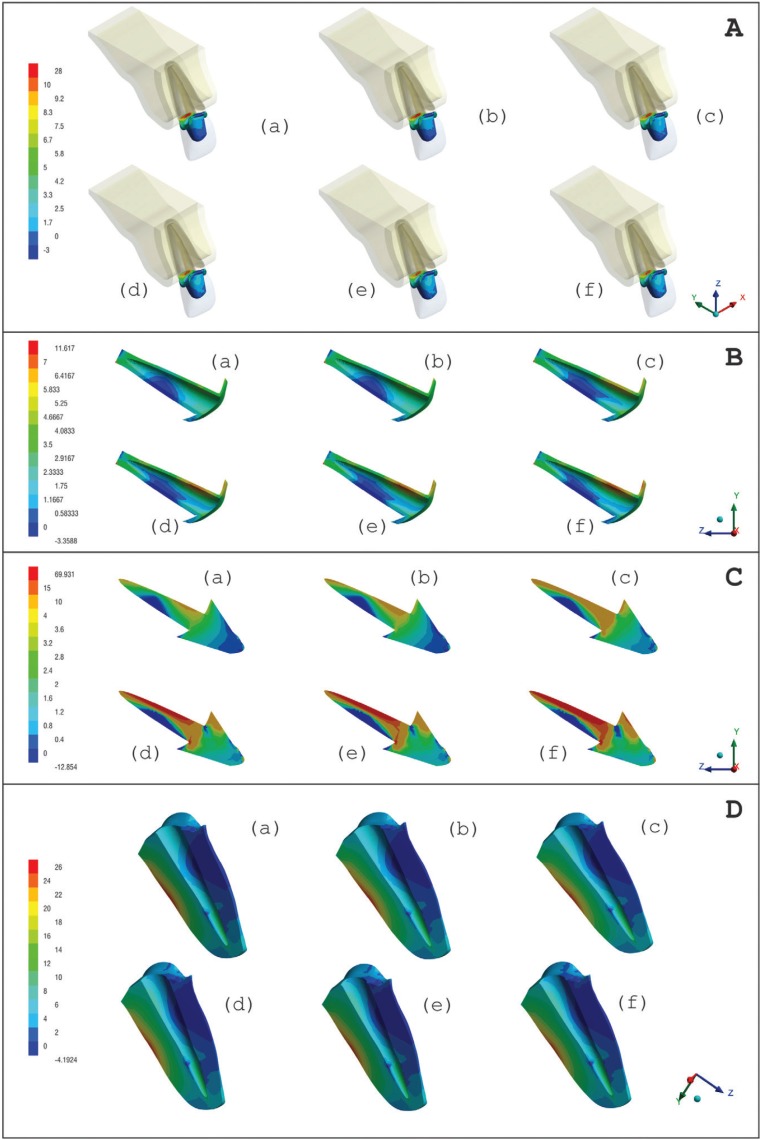
MPS distribution on the A) cement line between crown-core, B) cement line between post and root dentin, C) post-and-core and D) in dentin according to restorative materials: a) Nanoceramic resin, b) Composite resin, c) Hybrid ceramic, d) Lithium disilicate, e) Titanium, f) Zirconia Y-TZP.

**Figure 3:**
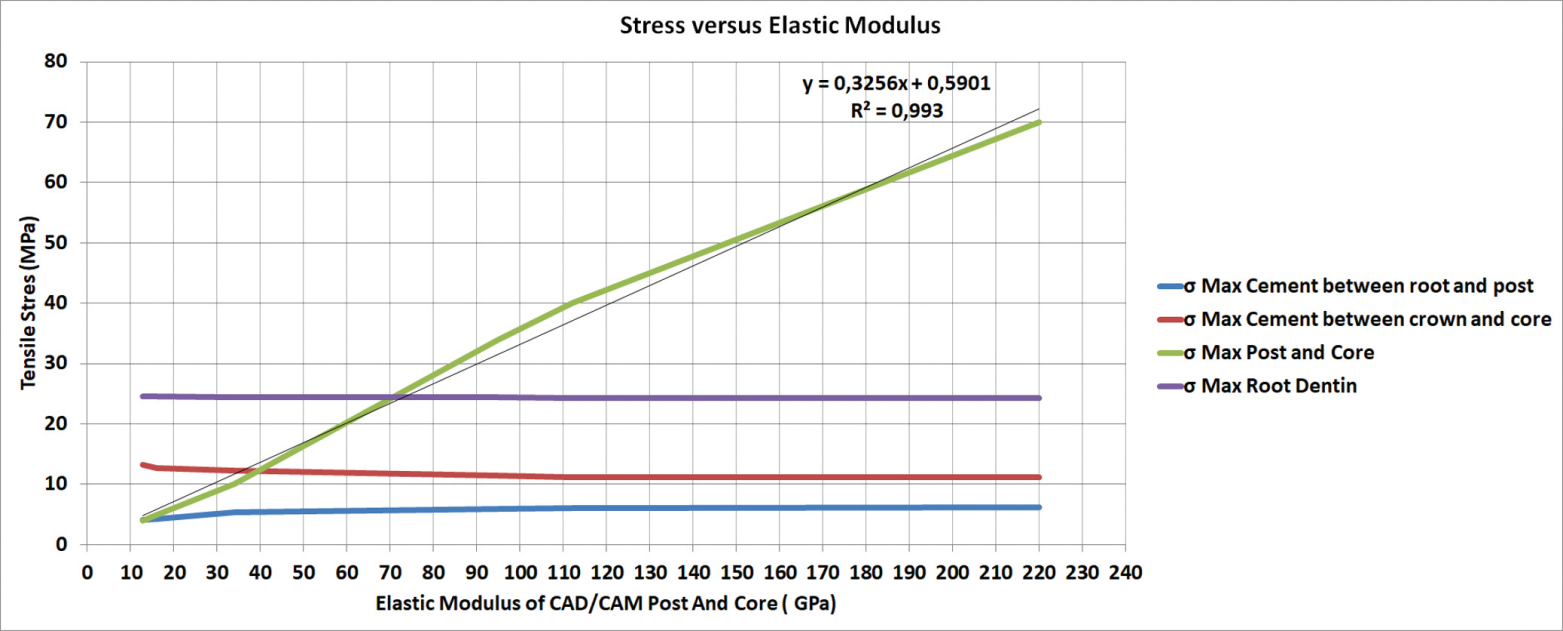
Line graph of the variation according to the tensile stress concentration as a function of the elastic modulus of the post and core.

 Table 2 shows the maximum tensile stress peaks in each region obtained for all groups. Maximum Principal Stress (MPS) analysis demonstrated that higher stress concentration in the cement line between crown-core when a post and core with lower elastic modulus was used (Fig. 2A). On the other hand, stiffer post and core showed more stress concentration in the cement line between post and dentin (Fig. 2B). However, the difference between groups was only 2 MPa for the crown/core and post/dentin cement lines. More homogeneous distribution (tensile stress) in the post area was observed when using materials with elastic modulus closer to dentin (nanoceramic resin and composite resin) (Fig. 2C). Regardless of post-and-core material, stress distribution in dentin was similar for all groups (Fig. 2D). Figure 3 shows a linear graph of the tensile stress concentration versus the elastic modulus of the post and core, corroborating that the higher the elastic modulus of the post and core the higher the stress concentration on its structure.

**Table 2:**
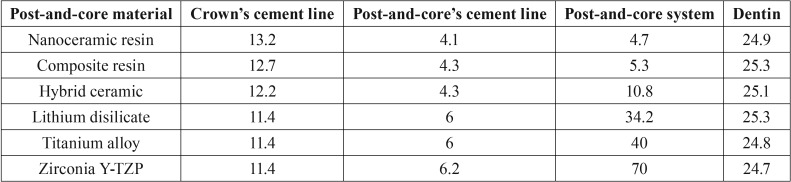
Maximum tensile stress peaks (MPa) in each region obtained for all groups.

## Discussion

The characteristics of the interfaces and the rigidity of the materials strongly influence the biomechanical behavior of teeth treated with post and cores (14). In addition, failure modes observed in the oral cavity usually occur during functional or parafunctional cyclic loads, repeated over a long period of time; failures by maximum stress would occur in situations such as dental trauma or during removal of temporary crowns (18). Even though finite element analysis is a static and linear analysis, this computational study can predict specific results, including mechanical behavior of the materials (19), mainly because fatigue failures begin in areas of stress concentration (20).

The first hypothesis was rejected, since the results showed a higher stress concentration in the crown cementation line when using post-and-core technique with lower elastic modulus (Fig. 2A). As in the study by Kelly *et al.* (21) and Dejak and Młotkowski (22), ceramics cemented under rigid substrates such as cast metal cores exhibit longer survival time and lower failure rate because the tensile stress concentration is lower than those cemented in dentin. However, our current study demonstrated that the maximum tensile stress difference between the less and more rigid material was approximately 2 MPa ( Table 2), and did not show a significant difference for the bond strength of the crown. However, this stress would possibly be relevant when the individual bond strength of each material were taken into account. In this sense, the results show that the maximum stress distribution exceeded the bond strength ( Table 3) (10,22-29) of zirconia with surface treatment (7.9 ± 2.6 MPa) (26), and was close to the resistance of titanium (14.79 ± 2.33 MPa) (27) and the nanoceramic resin (14.35 ± 2.56) (25).

**Table 3:**
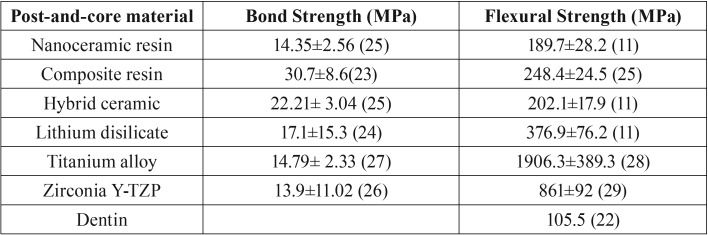
Mean values of bond strength (MPa) between resin cement and post-and-core material and mean values of flexural strength (MPa) of each material used in the study.

Moreover, the our results showed that lower elastic modulus material generates lower concentration stress at the cement line between the post and dentin, compared with a post-and-core system made with more rigid materials (Fig. 2B), thus rejecting the second hypothesis. The lowest stress concentrations were in the posts made of nanoceramic resin, hybrid ceramic and composite resin ( Table 2). Therefore, restorative materials with low and unstable resin bond strength might have a higher displacement risk failure. As it is already known, the high C factor, presence of humidity, lower light penetration of the curing lights, access to intracanal dentin walls for adhesion procedures make the adhesion to root dentin difficult (30).

An accumulation of flaws combined with cyclic loads concentrates stresses at the adhesive interfaces, thus causing fatigue in the materials even if the forces do not reach the tensile limit (31). Bottino *et al.* (32) demonstrated that after mechanical cycling, zirconia posts cemented with resin luting agents had their bond strength decreased in root dentin. This probably occurred due to the high elastic modulus of the material that generates a higher stress concentration in the cementation line, in addition to the lower adhesive strength of the polycrystalline zirconia, which increased the fatigue risk at the interface during the load incidence. In contrast, less rigid materials would suffer less fatigue effect on their bond strength, since the stress distribution at this interface is more homogeneous (30), so we could assume that posts with smaller elastic modulus would have less displacement risk.

The third null hypotheses was rejected in this study, because the stress distribution in the post was inversely proportional as the elastic modulus of the materials increased (Fig. 2C; Fig. 3) (more rigid material generated 15 times higher stress) ( Table 2). The systems made of zirconia and titanium had the highest stress concentration at the post, with a maximum tensile stress concentration of 40 and 70 MPa, respectively ( Table 2). However, the stress peak would not reach the flexural strength of these materials (28,29) (Table 3), therefore, failure by post fracture would not be expected. Another material that obtained higher values of stress concentration was lithium disilicate with around 34 MPa ( Table 2), lower than its flexural strength, around 376 MPa (11) ( Table 3). However, it is important to highlight the fact that ceramic materials fail in the oral environment with lower loads than those obtained through fatigue resistance tests (10). The complex combination of cyclic loads, the presence of water and the alternation of thermal and chemical conditions in the oral environment result in a decrease in the resistance of the ceramics, leading to failure (21). Also, damages can be intensified using the sliding contact during load application (32,33). This can be attributed to different microstructures, meaning the variation of the ratio between glassy and crystalline phase strongly influences the propagation of cracking and its mechanical properties. Because lithium disilicate is composed of a glassy phase, it has a higher risk of failure when compared to a polycrystalline ceramic such as zirconia (34), suggesting that it would not be suitable for making intrarradicular post-systems even with lower tensile stress concentration (Fig. 2C,D).

A new class of dental material was evaluated in this study: a polymer-infiltrated ceramic (hybrid ceramic) with a microstructure containing 86% (by weight) porous feldspar ceramic matrix infiltrated with a copolymer (urethane dimethacrylate and triethylene glycol dimethacrylate) (16). Although there was no studies indicating or having performed a post-and-core system with this material, we observed that the maximum tensile stress at the post was 10 MPa, while the flexural strength value of this material in a laboratorial study was 202 MPa (11) ( Table 3), so it can be assumed that the material would not fracture. Even though hybrid ceramics have a high glass matrix content, which under fatigue would increase the risk of failure, this new material has a mechanism that limits the crack propagation due to the presence of the two phases (35). Therefore, the material in our study would have potential for use in one-piece cast-and-post system.

Finally, the fourth null hypothesis was not rejected because the tensile stress distribution in dentin was similar for all the type of materials (Fig. 2D;  Table 2), not reaching the flexural strength of dentin ( Table 3). This probably occurred because the luting agent has an elastic modulus similar to dentin, and this layer between the dentine and the post would reduce the stress concentration in the dentin (19). Less rigid posts allow the teeth to bend when subjected to loads, providing better stress distribution at the post/cement/dentin interface (36). This can influence the failure mode of the weakened remnant. A laboratorial study (37) observed that endodontically treated teeth restored with zirconia posts systems had higher rates of catastrophic failure. In that study, less rigid posts such as fiberglass posts showed adhesive failure before the occurrence of root/post/core fracture. This may be positive for the remaining tooth, since failure of the post prior to the remnant would preserve the teeth from catastrophic failure, but it can compromise the prosthetic assembly.

Although the finite element analysis is a reliable method to predict potential failures (19), the limitation of this method is that the materials and structures are considered linearly elastic and homogeneous, which does not occur in real conditions. Also, the mean masticatory load in anterior region can vary (per exemple from 93N to 206N) (38) according to gender (38-40), age (39), race (38), cranio-facial morphology, periodontal support of teeth, temporomandibular disorders and pain (41), weight and height (40) and dental absence (40,42). In addition, as already discussed, failure in an oral environment normally occurs due to fatigue and slow crack growing (20). Therefore, in order to evaluate the behavior of these materials under fatigue, more laboratory and clinical studies should be conducted to better predict the success of these new treatment proposals evaluated by this current investigation.

From this study, the following can be concluded:

The elastic modulus of the post and cores made by CAD/CAM facility are directly proportional to the concentration of deleterious stress in its own structure. Thus, it suggests a better prognosis of stress distribution to the luting cement and the root dentin with the use of materials with lower elastic modulus.
